# Global Health Estimate of Invasive *Mycobacterium chimaera* Infections Associated with Heater–Cooler Devices in Cardiac Surgery

**DOI:** 10.3201/eid2403.171554

**Published:** 2018-03

**Authors:** Rami Sommerstein, Barbara Hasse, Jonas Marschall, Hugo Sax, Michele Genoni, Matthias Schlegel, Andreas F. Widmer, the Swiss Chimaera Taskforce

**Affiliations:** Bern University Hospital, Bern, Switzerland (R. Sommerstein, J. Marschall);; University Hospital Zurich, Zurich, Switzerland (B. Hasse, H. Sax, M. Genoni);; Triemli City Hospital, Zurich (M. Genoni);; Cantonal Hospital St. Gallen, St. Gallen, Switzerland (M. Schlegel);; Basel University Hospital, Basel, Switzerland (A.F. Widmer)

**Keywords:** *Mycobacterium chimaera*, endocarditis, cardiac surgery, prosthetic valve, surgical site infection, incidence, prevalence, heater–cooler devices, global health, Switzerland, bacteria, tuberculosis and other mycobacteria

## Abstract

Investigations of a worldwide epidemic of invasive *Mycobacterium chimaera* associated with heater–cooler devices in cardiac surgery have been hampered by low clinical awareness and challenging diagnoses. Using data from Switzerland, we estimated the burden of invasive *M. chimaera* to be 156–282 cases/year in 10 major cardiac valve replacement market countries.

Invasive *Mycobacterium chimaera* infection associated with heater–cooler devices (HCDs) in cardiac surgery was identified as a new disease entity in 2014 (*1*). The most likely pathogenesis involves aerosols transmitted from the HCD to the patient during surgery (*2*). As of September 2017, ≈120 cases have been recognized globally; mycobacterial device contamination at the manufacturing site of the LivaNova 3T (LivaNova, London, UK), the HCD market leader, seems to represent the most likely point source for the outbreak (*3*,*4*). The incubation period after exposure is long, with a median of 17 months (range 3–72 months). Signs and symptoms are generally nonspecific and include fatigue, fever, and weight loss. There is no established therapy, and the case-fatality rate is ≈50% (*3*,*5*). The disease appears to be rare and most commonly affects patients after valve replacement or other implant procedures in open heart surgery. Currently, the extent of the epidemic is unknown. We aimed to estimate the global epidemiology of disseminated *M. chimaera* disease associated with open heart surgery. The study followed the Guidelines for Accurate and Transparent Health Estimates Reporting (*6*).

## The Study

### Case Definition 

Switzerland was the leading country in recognizing and researching the global outbreak of *M. chimaera* disease associated with open heart surgery (*1*,*2*,*5*,*7*,*8*). This recognition included early establishment of a nationwide interdisciplinary expert group consisting of hospital epidemiologists, infectious disease physicians, cardiac surgeons, perfusionists, microbiologists, and consultants from the Swiss Agency for Therapeutic Products and the Federal Office of Public Health (collectively called the Swiss Chimaera Taskforce). This expert group has issued nationwide recommendations on case definition, lookback mechanisms, and infection prevention measures and releases updates on proven cases (*9*,*10*). We identified cases for our study through review of clinical and microbiological data, based on a previously published case series (*7*) and on reports to the Swiss Chimaera Taskforce. A proven case was defined as a case in a patient with previous open heart surgery, including implant surgery, and the subsequent detection of the *M. chimaera* outbreak strain from a sterile site.

### Valve Replacements and HCDs in Use

Data on annual valve replacements performed during 2008–2016 were available from the Swiss National Registry for Cardiac Surgery. We report aggregated data for mitral and aortic valves. Currently active HCDs with the reporting date of January 15, 2015, were submitted via an electronic spreadsheet to the Swiss Chimaera Taskforce.

### Statistical Analyses

We obtained data on demographic development from the Swiss Federal Office of Statistics (*11*). By applying a linear regression model, we determined estimates of the annual prevalence per 1,000 valve replacement procedures for index surgeries from 2008 (the presumed beginning of the outbreak) through 2014 (taking the latency of disease manifestation into account). Likewise, we calculated the yearly incidence of detected cases per 1,000,000 inhabitants from 2011 (the year of the first diagnosed case) through 2016. We determined best fit values and 95% CIs.

We identified 11 proven cases during January 2008–September 2017 (Figure, panel A). The number of annual valve replacement procedures increased from 1,632 in 2008 to 2,581 in 2016. During the same period, the population of Switzerland increased from 7,593,494 to 8,327,126 (Table). The annual estimated incidence of proven cases per 1 million inhabitants increased from 0.16 (95% CI 0–0.37) in 2011 to 0.25 (95% CI 0.05–0.45) in 2016 (p = 0.49). The prevalence per 1,000 valve replacement surgeries increased from 0.52 (95% CI 0–1.41) in 2008 to 0.94 (95% CI 0.05–1.83) in 2014 (p = 0.49; Figure, panel B). The LivaNova 3T was the predominant HCD, in use by 15 of 18 reporting cardiac surgery centers in Switzerland as of January 15, 2015, with 28 of 44 HCDs in use (64%). This rate is comparable with the worldwide LivaNova 3T market share of 70% (*3*).

**Figure:**
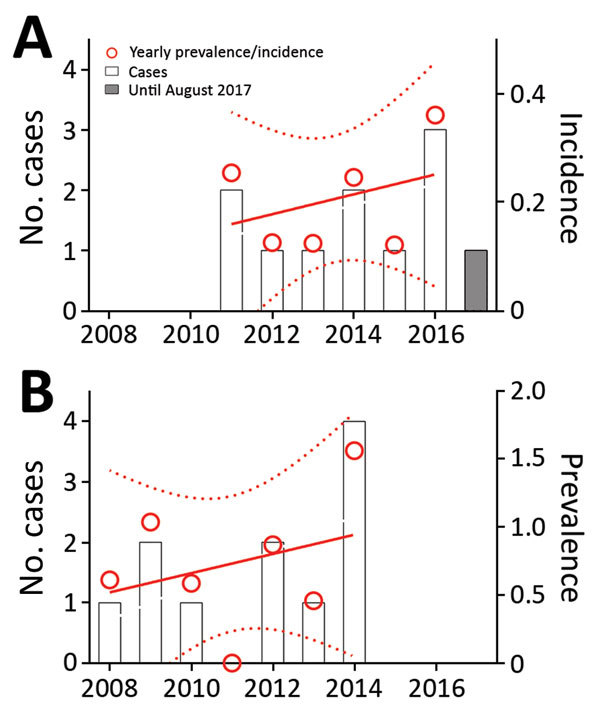
Incidence and prevalence of invasive *M. chimaera* cases in Switzerland since 2008. A) Yearly incidence per 1 million inhabitants according to the date of detection. B) Prevalence per 1,000 valve replacement surgeries according to the date of index sugery. Numbers of cases are projected on the left *y*-axis (bars); prevalence and incidence on the right *y*-axis (red circles). Gray bar indicates no. cases for January–August 2017. Linear regressions (red lines) were modeled for the respective window periods of interest; dotted lines indicate 95% CIs.

**Table:** Numbers of *Mycobacterium chimaera* case), annual valve replacement procedures, and population in Switzerland, 2008–2017

Year	Cases by date of index surgery	Cases by date of detection	No. aortic valves	No. mitral valves	Total no. valves	Total population
2008	1	0	1,291	341	1,632	7,593,494
2009	2	0	1,238	691	1,929	7,701,856
2010	1	0	1,306	398	1,704	7,785,806
2011	0	2	1,468	274	1,742	7,870,134
2012	2	1	2,032	273	2,305	7,954,662
2013	1	1	2,017	164	2,181	8,039,060
2014	4	2	2,120	441	2,561	8,139,631
2015	0	1	2,039	472	2,511	8,237,666
2016	0	3	2,112	469	2,581	8,327,126
2017	0	1	NA	NA	NA	NA

*NA, not available.

The prevalence we found is similar to that in a previously published report of a relatively crude estimation (*12*) and about 4 to 7 times higher than a UK study based on national laboratory and hospital admissions data (*13*). The difference from the UK study can be explained by the active case detection strategy in Switzerland, compared with the laboratory investigation in the United Kingdom (*10*,*13*).

## Conclusions

The risk for *M. chimaera* in patients undergoing heart surgery is similar to the risks for more widely known diseases such as parathyroid carcinoma (0.2/million/y), adrenal carcinoma (0.3/million/y), and congenital rubella syndrome (0.3/million/y) (*14*) that draw more attention but may have a smaller preventable proportion.

Considering an estimated 300,000 global annual valve replacement surgeries in the 10 major market countries and a US population of 323 million (*12*,*15*), we can extrapolate our findings to an annual incidence of 156–282 cases for the 10 major valve replacement markets and 51–80 cases in the United States alone. The currently known cases of invasive *M. chimaera* disease related to valve replacement reported from around the globe (n ≈ 120) are well below our estimate (*3*,*4*). Many countries in Europe did not report cases during the past few years, which is likely due to underreporting or underdiagnosing (*3*,*4*).

The study has limitations. Invasive *M. chimaera* is not a mandatory reportable disease in Switzerland. However, the small size of the country and the close collaboration of the Swiss Chimaera Taskforce with the professional societies, as well as the broad coverage of the outbreak in television and newspapers, decreases the likelihood of underreporting. In addition, the Swiss National Center for Mycobacteria in Zurich receives most, if not all, strains for confirmation. As demonstrated previously, some patients may still be misinterpreted as having a rheumatologic disease (*7*), a potential reason for reporting bias. With valve replacement surgery as the denominator, we may overestimate the incidence/prevalence of invasive *M. chimaera*, as it is possible that some cases resulted from a procedure other than valve replacement surgery. All cases in Switzerland were associated with valve replacement surgery, however. Furthermore, many factors may influence incidence/prevalence on a global level, such as the predominant HCD brands in use, the degree of mycobacterial contamination of these HCDs, the built hospital environment, positioning in the operating room, and the number and type of cardiac surgery procedures performed. Infection after HCD exposure is likely to be unevenly distributed and may be quite high in 1 hospital but near zero in a nearby hospital, even though each hospital uses the implicated HCD. It is essential to investigate this variability and understand the relative contribution of some of these variables to the risk of infection. Nevertheless, it is likely that the aggregated risk derived from these factors is similar across national healthcare systems.

In summary, our data provide an estimate of the global burden of *M. chimaera* associated with open heart surgery, enabling policy makers to guide actions and to decrease the risk for transmission from HCDs. Our data suggest implementation of systematic lookback approaches in each country where LivaNova 3T HCDs have been used to optimize case finding. In addition, countries may consider mandatory reporting of invasive nontuberculous mycobacterial infections.

## References

[1] Sax H, Bloemberg G, Hasse B, Sommerstein R, Kohler P, Achermann Y, et al. Prolonged outbreak of *Mycobacterium chimaera* infection after open-chest heart surgery. Clin Infect Dis. 2015;61:67–75. 10.1093/cid/civ198 25761866

[2] Sommerstein R, Rüegg C, Kohler P, Bloemberg G, Kuster SP, Sax H. Transmission of *Mycobacterium chimaera* from heater-cooler units during cardiac surgery despite an ultraclean air ventilation system. Emerg Infect Dis. 2016;22:1008–13. 10.3201/eid2206.160045 27070958 PMC4880077

[3] Walker J, Moore G, Collins S, Parks S, Garvey MI, Lamagni T, et al. Microbiological problems and biofilms associated with *Mycobacterium chimaera* in heater-cooler units used for cardiopulmonary bypass. J Hosp Infect. 2017;96:209–20. 10.1016/j.jhin.2017.04.014 28532976

[4] van Ingen J, Kohl TA, Kranzer K, Hasse B, Keller PM, Katarzyna Szafrańska A, et al. Global outbreak of severe *Mycobacterium chimaera* disease after cardiac surgery: a molecular epidemiological study. Lancet Infect Dis. 2017;17:1033–41. 10.1016/S1473-3099(17)30324-9 28711585

[5] Sommerstein R, Schreiber PW, Diekema DJ, Edmond MB, Hasse B, Marschall J, et al. *Mycobacterium chimaera* outbreak associated with heater-cooler devices: piecing the puzzle together. Infect Control Hosp Epidemiol. 2017;38:103–8. 10.1017/ice.2016.283 27839530

[6] Stevens GA, Alkema L, Black RE, Boerma JT, Collins GS, Ezzati M, et al.; The GATHER Working Group. Guidelines for Accurate and Transparent Health Estimates Reporting: the GATHER statement. Lancet. 2016;388:e19–23. 10.1016/S0140-6736(16)30388-9 27371184

[7] Kohler P, Kuster SP, Bloemberg G, Schulthess B, Frank M, Tanner FC, et al. Healthcare-associated prosthetic heart valve, aortic vascular graft, and disseminated *Mycobacterium chimaera* infections subsequent to open heart surgery. Eur Heart J. 2015;36:2745–53. 10.1093/eurheartj/ehv342 26188001

[8] Schreiber PW, Kuster SP, Hasse B, Bayard C, Rüegg C, Kohler P, et al. Reemergence of *Mycobacterium chimaera* in heater-cooler units despite intensified cleaning and disinfection protocol. Emerg Infect Dis. 2016;22:1830–3. 10.3201/eid2210.160925 27649345 PMC5038437

[9] Federal Office of Public Health, Bern, Switzerland. Richtlinien zum Betrieb und zur Ueberwachung von Heater-Cooler Devices (HCDs) im Operationssaal. 2017 Feb 9 [cited 2017 Apr 26]. https://www.bag.admin.ch/dam/bag/de/dokumente/mt/i-und-i/healthcare-assoziierte-infektionen/richtlinien-hcds-im-op-m-chimaera.pdf.download.pdf/richtlinien-hcds-im-op-mycobacterium-chimaera.pdf

[10] Hasse B. Potentiell nosokomiale Ansteckung mit *Mycobacterium chimaera*. Schweizerische Ärztezeitung. 2017;98:03. 10.4414/saez.2017.05271

[11] Federal Statistical Office, Bern, Switzerland. Population [cited 2017 May 29]. https://www.bfs.admin.ch/bfs/en/home/statistics/population.html

[12] Centers for Disease Control and Prevention. CDC advises hospitals to alert patients at risk from contaminated heater-cooler devices used during cardiac surgery. 2016 [cited 2017 May 23]. https://emergency.cdc.gov/han/han00397.asp

[13] Chand M, Lamagni T, Kranzer K, Hedge J, Moore G, Parks S, et al. Insidious risk of severe *Mycobacterium chimaera* infection in cardiac surgery patients. Clin Infect Dis. 2017;64:335–42. 10.1093/cid/ciw754 27927870 PMC5881635

[14] Orphanet Report Series. Prevalence of rare diseases: bibliographic data. November 2016 [cited 2017 Apr 27]. http://www.orpha.net/orphacom/cahiers/docs/GB/Prevalence_of_rare_diseases_by_alphabetical_list.pdf

[15] GlobalData. MediPoint. Prosthetic heart valves—global analysis and market forecasts. 2015. [cited 2017 May 29]. https://www.marketresearch.com/product/sample-8987879.pdf

